# First-trimester medication abortion via telemedicine: A retrospective cohort study

**DOI:** 10.1016/j.puhip.2024.100539

**Published:** 2024-08-23

**Authors:** Leonardo Cely-Andrade, Luis Carlos Enríquez-Santander, Karen Cárdenas-Garzón, Biani Saavedra-Avendaño, Guillermo Antonio Ortiz Avendaño

**Affiliations:** a Profamilia, Bogotá, Colombia; b Ipas Latin America and the Caribbean, Mexico City, Mexico; c Ipas Latin America and the Caribbean, North Carolina, United States

**Keywords:** Cohort study, Medication abortion, Telemedicine, Effectiveness, Safety, Satisfaction

## Abstract

**Background:**

Following the decriminalization of abortion in Colombia and amidst a global health crisis due to COVID-19, Profamilia implemented a telemedicine-assisted first-trimester Medication Abortion (MAB) program. This is an opportunity to reduce inequalities in access and to promote empowerment and sexual and reproductive rights. This study aims to describe socio-demographic and clinical characteristics of users and to assess its effectiveness and safety.

**Study design:**

A retrospective cohort study.

**Methods:**

The study analyzed data from users who received Profamilia's telemedicine abortion services between August 2021 and August 2022 (n = 3073). A descriptive analysis of their sociodemographic and clinical characteristics was performed, grouping, and comparing them according to follow-up status and abortion outcome. Effectiveness was assessed by the percentage of complete abortions without surgical intervention, and safety by the incidence of complications, potential adverse events, and potentially dangerous signs.

**Results:**

Most of the users were less than 8 weeks gestation at the start of treatment (88.3 %), from low socioeconomic strata (84.8 %), affiliated to the subsidized healthcare system (87.6 %), with educational levels up to secondary school (81.6 %), between 18 and 35 years (87.4 %), from urban areas (97.8 %) and singles (90,8 %). 94.9 % of users had a complete abortion using medication, and 0.3 % of cases reported complications.

**Conclusions:**

First-trimester MAB through telemedicine in the Latin American context is an effective and safe choice. Telehealth is an important strategy to expand access to safe abortion care, especially for those with limited financial means or educational backgrounds. Rural and marginalized populations need more attention to improve access.

## Research in context

**Evidence before this study:** Prior to the analysis conducted in this study, we performed a systematic literature review on telemedicine medication abortion up to the twelfth week of pregnancy. The search was conducted in January 2023 on the PubMed, Embase, Cochrane, SciELO, LILACS, and Google Scholar databases. It was based on the Population, Intervention, Comparison, Outcomes, and Study Design (PICOS) framework. Also, the search was not restricted to any particular year of publication, and studies could be published in English or Spanish. Study screening and selection, risk of bias assessment, and data extraction were performed by peer reviewers. A total of 21 articles published between 2011 and 2022 met the inclusion criteria: twenty were observational studies, and one was a randomized clinical trial. Regarding the risk of bias, five studies had a serious risk, fifteen had a moderate risk, and one had an undetermined risk. Concerning the type of intervention, seven studies compared telemedicine with standard care. When examining effectiveness by gestational week subgroups using risk difference (RD), the meta-analysis revealed a slight advantage of telemedicine over in-person care: for pregnancies up to the ninth week (RD = 0.01; 95 % CI – 0.00, 0.03); for pregnancies up to the tenth week (RD = 0.01; 95 % CI – 0.00, 0.01); and for pregnancies up to the twelfth week (RD = 0.01; 95 % CI – 0.01, 0.02). With respect to the other meta-analyses, no differences were observed in the occurrence of adverse events or satisfaction with the service when comparing the two forms of healthcare delivery.

**Added value of this study:** No studies have been conducted on telemedicine medication abortion in Colombia, and scientific evidence on the subject is scarce for Latin America.

**Implications of all the available evidence:** By characterizing users of telemedicine medication abortion services, it is possible to gather valuable information to improve and modify healthcare services, thus satisfactorily responding to women's sexual and reproductive health needs.

## 1 Introduction

Universal access to sexual and reproductive health services is essential, as stated in the Universal Declaration of Human Rights and other international and regional conventions founded on the principles of equality and non-discrimination [1]. In certain regions, these principles are still in the early stages of implementation. In nations with unfavorable conditions, pregnant women seeking these services must resort to clandestine institutions, increasing the risk of complications and death [2]. The World Health Organization (WHO) has urged member countries to develop policies for the decriminalization of abortion and issued recommendations for healthcare systems to prioritize safe abortion services [1,[3], [4], [5], [6]].

Unsafe abortion is the termination of a pregnancy by individuals lacking sufficient technical knowledge or under conditions that do not meet the minimal medical standards [7]. It is a determinant of maternal morbidity and mortality, claiming the lives of around 47,000 pregnant women worldwide each year [1,6]. Approximately 60 % of unsafe abortions occur in Africa, and 30 % in Latin America [8]. This lack of access to safe, timely, and affordable abortion services increases the risk of health complications for pregnant women and imposes costs on healthcare services, making it a human rights and public health concern [9].

Currently, more countries are decriminalizing abortion. In Colombia, public health policies and laws have been modified to comply with international guidelines. Abortion in Colombia is legal and covered by the health system in cases of rape, risk to the mother's health, and if extrauterine life is not possible. As of 2022, it's legal until the 24th week of gestation for any reason [10,11].

MAB up to the twelfth week of pregnancy has been a significant milestone due to the simplicity and safety of the procedure. This treatment is available in healthcare institutions, or it can be self-managed at home [1]. The outpatient option frees users from the structural limitations of healthcare services [12] and opens possibilities for innovative care alternatives, such as the use of telemedicine services [13,12].

Telemedicine was crucial during the COVID-19 pandemic, helping healthcare systems adapt to mobility restrictions and lockdown measures. It reduced geographical barriers, improved care quality and timeliness, optimized costs, and reduced waiting times [[14], [15], [16], [17], [18], [19], [20]]. In sexual and reproductive health, telemedicine can be used for preventive care, contraceptive counselling, family planning, infertility studies, MAB, etc. [18].

Colombia's healthcare system is regulated by Law 100 of 1993. Higher income earners contribute a larger proportion to healthcare to support those with limited resources. For this reason, there are two affiliation regimes: contributory (workers and their families) and subsidized (unemployed people). There is also a third special scheme, which protects officials of government institutions, the armed forces and ministries, among others [21]. In all the regimes, health services are provided through outsourcing, for example: Profamilia.

Several health administrators have partnered with Profamilia, a Colombian non-governmental organization, and its core mission is to advocate for the respect and exercise of Colombians’ sexual and reproductive rights. In 2021, powered by scientific findings and technological changes, Profamilia launched the *Mi cuerpo, Mi autonomía-MIA* (My Body, My Autonomy) program. It provides professional guidance to pregnant women seeking voluntary termination of pregnancy up to the twelfth week through self-managed MAB. Telemedicine approach is adopted for this service model, which has demonstrated effectiveness and safety on par with or better than in-person care [[22], [23], [24], [25]].

This study aims to characterize the users of the MIA program and to evaluate the safety and effectiveness of MAB through telemedicine in Colombia, a country with high wealth inequality and limited healthcare access for rural residents, ethnic groups, migrants, and those in the subsidized healthcare system [26].

## 2 Methods

### 2.1 Study design and participants

We conducted a retrospective cohort study including all pregnant women who requested telemedicine abortion services within the first trimester of pregnancy through the Profamilia MIA program in Colombia, from August 1st, 2021, to August 31st, 2022.

The MIA program provides services throughout Colombia. It can be paid for by health insurers or by the client. Pregnant women may contact MIA staff at any time of the day, seven days a week, via the website, telephone, or in-person [11]. Physicians assesses user's gestational weeks based on their Last Menstrual Period (LMP) to determine their eligibility for MAB. If the LMP is not reliable, an ultrasound or a blood test is advised to estimate gestational length.

During the telemedicine consultation, physician prescribes the MIA kit, which includes one tablet of mifepristone 200 mg for oral administration and four tablets of misoprostol 200 mg for sublingual administration 36 h after, in accordance with the WHO's guidelines for MAB [27]. Additionally, the kit contains a urine pregnancy test, tablets of ibuprofen 400 mg, a condom, and oral contraceptives for 21 or 28 days. Users are informed about potential side effects, warning signs, and necessary visits to the emergency department, as well as contraceptive methods.

### 2.2 Data sources

During the initial consultation with the physician, sociodemographic data, clinical and obstetrical history, and clinical information regarding the user's gestational status were recorded.

The follow-up calls to establish treatment success or failure and secondary adverse events was made 21 days after the start of treatment. A satisfaction survey was also sent via e-mail which could be answered on a voluntary basis.

The effectiveness of MAB was obtained by a rapid urine pregnancy test performed 15 days after initiation of treatment. A negative result was interpreted as a successful abortion and the user was referred to a family planning clinic. Otherwise, the individual was advised to go to Profamilia for an in-person consultation to decide whether to continue with the pregnancy or to resort to an extra dose of misoprostol or manual vacuum aspiration.

### 2.3 Data collection

Data were obtained from Profamilia's Health Information Systems database. Each record underwent data quality control, and the completeness of the variables was also validated.

### 2.4 Variables

The sociodemographic variables included age, area of residence, city, socioeconomic status, level of education, healthcare affiliation regime, marital status, and ethnic minority group. The clinical variables included gestational weeks at the time of the first consultation, and number of previous pregnancies.

The *age* variable was presented as continuous and divided into four groups: <18, 18–24, 25–34, and ≥35. The six socioeconomic strata were condensed into three groups: low [1,2], middle [3,4], and high [5,6]. Ethnicity was classified into two categories: none and others (indigenous, Afro-descendant, Raizal, Palenque, and Rom). Regarding marital status, participants who identified themselves as separated, divorced, or widowed were grouped with those in the “single” category, while those in de facto unions were grouped with those in the “married” category. Gestational weeks were divided into two categories: less than or equal to eight weeks and more than eight weeks. This was done because kit delivery and medication intake could take up to three weeks, which could exceed the twelve-week threshold recommended for MAB. The location of the users was classified as urban or rural in accordance with the classification of the National Administrative Department of Surveys (DANE) [28].

### 2.5 Results definition

All data collected were self-reported by users, except for gestational weeks, which were estimated through blood tests or ultrasound for some observations.

Successful abortion was defined according to the Medical Abortion Reporting of Efficacy (MARE) guidelines, which define it as the “successful expulsion of the intrauterine pregnancy without need for surgical intervention” [29].

Safety results were categorized into three groups: potentially dangerous signs (excessive bleeding, pain not relieved by analgesics, fever greater than 38 °C, and signs suggestive of local infection of the reproductive system), potentially adverse events (requirement of intravenous fluids, blood transfusion and hospitalization) [30] and complications (infection, hemorrhage, uterine rupture or death) [31]. Additionally, symptoms such as pain, bleeding, nausea/vomiting, diarrhea, chills, and headache are described as common side effects.

Using a Net Promoter Score (NPS) of 0–10, users were evaluated on their satisfaction. An overall rating of 10 indicates absolute satisfaction, while a rating of 0 indicates the opposite. The users are classified according to their scores, with those who receive a score of 9 or 10 points being classified as service promoters and those who receive a score of 8 or less being classified as non-service promoters [32].

### 2.6 Statistical methods

The study compared sociodemographic and clinical variables among participants receiving MAB services, those without, and those providing follow-up information. Among participants with follow-up data, comparisons were made based on the MAB results. Since not all observations include satisfaction information, The sociodemographic characteristics of users who completed the satisfaction survey are compared to those who didn't. This was done to identify potential biases in the satisfaction results.

We conducted a geographical analysis to identify the main areas of residence of telemedicine service users. Based on this information, the departments with the highest number of users in the territory were identified. The free software EpiInfo V.7.2 was used for this purpose.

For qualitative variables, absolute frequencies and percentages were used and compared using the Chi-square test or Fisher's exact test. Quantitative variables were described using the median and interquartile range and compared using the Mann–Whitney *U* test due to their non-normal distribution. A significance level (α) of 0.05 was considered, and data were analyzed using the statistical software R (version 4.2.1).

## 3 Results

**Fig. 1:**
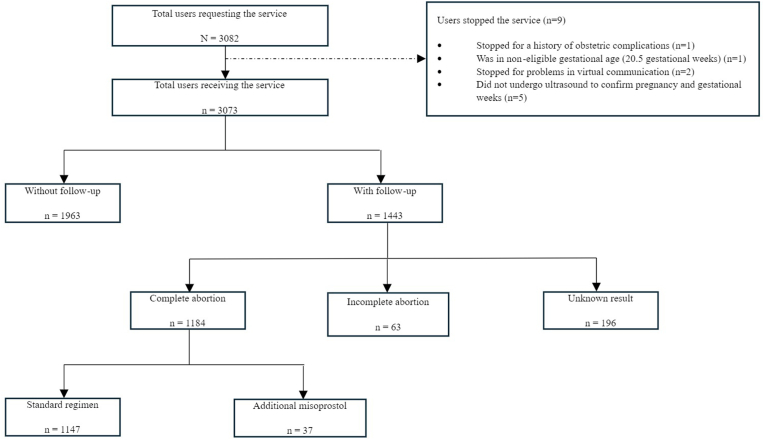
Flowchart of Profamilia's telemedicine medication abortion service, Colombia, 2021–2022.

3082 pregnant women requested telemedicine MAB services through the Profamilia MIA program between August 1st, 2021, and August 31st, 2022. Of these, nine (0.3 %) were not eligible for the procedure. Fig. 1 describes the reasons why the nine pregnant women were not eligible, the process of classifying clients throughout the study analysis, and the individuals who were not followed up effectively.

### 3.1 Care process

Eighty-seven per cent of users were aged between 18 and 35. The majority lived in urban areas (98 %), had a high school education (82 %), were covered by compulsory health insurance (72 %), were single (91 %) and had no ethnic affiliation (90 %). 88.3 % of clients started treatment before the 8th week of pregnancy and 49.9 % had a previous pregnancy (Table 1).

**Table 1:** Sociodemographic characteristics of Profamilia's telemedicine medication abortion service users, by follow-up status and medication abortion results, Colombia, 2021–2022.

Characteristics	Users who received the service				Abortion result			
	Total	With follow-up	Without follow-up	p-value	Total	Medication abortion	Procedural abortion	p-value
	n = 3073	n = 1443	n = 1630		n = 1247	n = 1184	n = 63	
	***n (%)***	***n (%)***	***n (%)***		***n (%)***	***n (%)***	***n (%)***	
Sociodemographic rowhead								
**Age**								
Min-Max	13–47	15–46	13–47	–	15–46	15–46	18–43	–
Median/[IQR]	25/[22–29]	25/[22–30]	25/[21–29]	**0.036**^a^	25/[22–30]	25/[22–29]	28/[24–32]	**0.002**^a^
<18 years	108 (3.5)	42 (2.9)	66 (4.0)	0.107^b^	38 (3.0)	38 (3.2)	0 (0.0)	**0.020**^b^
18–24 years	1326 (43.2)	605 (41.9)	721 (44.2)		529 (42.4)	510 (43.1)	19 (30.2)	
25–34 years	1359 (44.2)	654 (45.3)	705 (43.3)		564 (45.2)	531 (44.8)	33 (52.4)	
≥35 years	280 (9.1)	142 (9.8)	138 (8.5)		116 (9.3)	105 (8.9)	11 (17.5)	
**Area of residence**								
Urban	3004 (97.8)	1414 (98.0)	1590 (97.5)	0.780^b^	1223 (98.1)	1162 (98.1)	61 (96.8)	0.344^c^
Rural	64 (2.1)	29 (2.0)	35 (2.1)		24 (1.9)	22 (1.9)	2 (3.2)	
Missing	5 (0.2)	0 (0.0)	5 (0.3)	–	0 (0.0)	0 (0.0)	0 (0.0)	–
**Socioeconomic status**								
Low (strata 1 and 2)	2605 (84.8)	1203 (83.4)	1402 (86.0)	**0.026**^b^	1042 (83.6)	989 (83.5)	53 (84.1)	0.508^b^
Middle (strata 3 and 4)	449 (14.6)	230 (15.9)	219 (13.4)		198 (15.9)	189 (16.0)	9 (14.3)	
High (strata 5 and 6)	14 (0.5)	10 (0.7)	4 (0.2)		7 (0.6)	6 (0.5)	1 (1.6)	
Missing	5 (0.2)	0 (0.0)	5 (0.3)	–	0 (0.0)	0 (0.0)	0 (0.0)	–
**Level of education**								
Up to primary school	31 (1.0)	13 (0.9)	18 (1.1)	**0.006**^b^	10 (0.8)	9 (0.8)	1 (1.6)	0.465^b^
Up to secondary school	2507 (81.6)	1133 (78.5)	1374 (84.3)		995 (79.8)	949 (80.2)	46 (73.0)	
Two- or three-year associate degree	157 (5.1)	69 (4.8)	88 (5.4)		57 (4.6)	52 (4.4)	5 (7.9)	
Undergraduate/Postgraduate degree	151 (4.9)	90 (6.2)	61 (3.7)		79 (6.3)	75 (6.3)	4 (6.3)	
Missing	227 (7.4)	138 (9.6)	89 (5.5)	–	106 (8.5)	99 (8.4)	7 (11.1)	–
**Healthcare affiliation regimen**								
Subsidized	472 (15.4)	219 (15.2)	253 (15.5)	**<0.001**^b^	196 (15.7)	182 (15.4)	14 (22.2)	0.346^b^
Contributory	2220 (72.2)	988 (68.5)	1232 (75.6)		856 (68.6)	816 (68.5)	40 (63.5)	
Special regime	375 (12.2)	236 (16.4)	139 (8.5)		195 (15.6)	186 (15.7)	9 (14.3)	
Missing	6 (0.2)	0 (0.0)	6 (0.4)	–	0 (0.0)	0 (0.0)	0 (0.0)	–
**Marital status**								
Single/Separated/Divorced/Widowed	2790 (90.8)	1302 (90.2)	1488 (91.3)	0.197^b^	1302 (90.2)	1049 (91.5)	253 (85.5)	0.079^b^
Married/De facto union	278 (9.0)	141 (9.8)	137 (8.4)		141 (9.8)	98 (8.5)	43 (14.5)	
Missing	5 (0.2)	0 (0.0)	5 (0.3)	–	0 (0.0)	0 (0.0)	0 (0.0)	–
**Ethnicity**								
None	3043 (99.0)	1433 (99.3)	1610 (98.8)	0.596^b^	1239 (99.4)	1176 (99.3)	63 (100.0)	0.660^c^
Other (Afro-descendant, *Raizal*)	24 (0.8)	10 (0.7)	14 (0.9)		8 (0.6)	8 (0.7)	0 (0.0)	
Missing	6 (0.4)	0 (0.0)	6 (0.4)	–	0 (0.0)	0 (0.0)	0 (0.0)	–
**Clinical**								
**Weeks' gestation**								
Min-Max	2.5–12.4	2.6–10.0	2.5–12.4	–	2.6–10.0	2.6–10.0	3.0–8.5	–
Median [IQR]	6.4 [5.6–7.3]	6.3 [5.5–7.2]	6.4 [5.6–7.3]	**0.006**^a^	6.3 [5.5–7.2]	6.3 [5.5–7.2]	6.3 [5.4–7.1]	0.530^a^
≤8 weeks	2714 (88.3)	1305 (90.4)	1409 (86.6)	**<0.001**^b^	1128 (90.5)	1068 (90.2)	60 (95.2)	0.185^b^
>8 weeks	356 (9.6)	138 (9.6)	218 (13.4)		119 (9.5)	116 (9.8)	3 (4.8)	
**Previous pregnancies**								
Yes	1533 (49.9)	696 (48.2)	837 (51.3)	0.116^b^	586 (47.0)	552 (46.6)	34 (54.0)	0.296^b^
No	1511 (49.2)	729 (50.5)	782 (48.0)		646 (51.8)	617 (51.2)	29 (46.0)	
Missing	29 (0.9)	18 (1.2)	11 (0.7)	–	15 (1.2)	15 (1.3)	0 (0.0)	–

a Mann–Whitney *U* test.

b Chi-Square Test of Independence.

c Fisher's Exact test.

Out of the total participants who received MAB services, 1443 (46.9 %) provided follow-up information. Table 1 shows the characteristics of users with and without follow-up data.

Users without follow-up data had gestation exceeding eight weeks, lower socioeconomic status and be affiliated with subsidized healthcare. Those who provide follow-up info are more likely to have higher education.

**Fig. 2:**
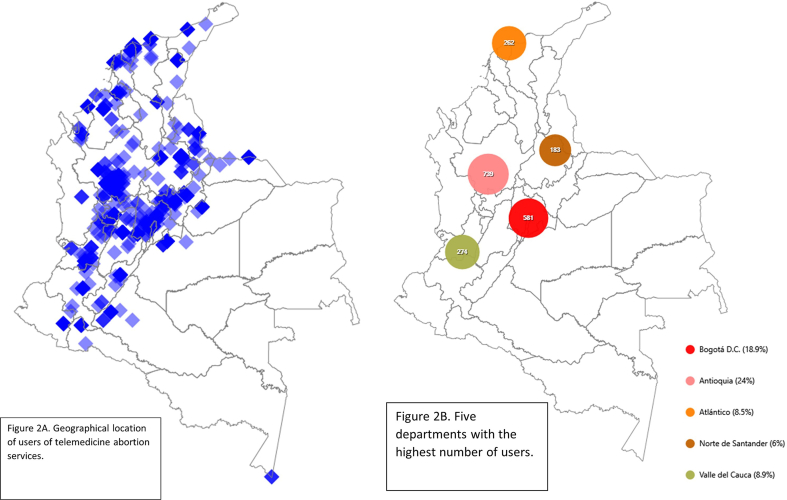
Figure 2A. Geographical distribution of MIA program 2021–2022 service users, Colombia. Figure 2B.The five departments with the highest number of users of MIA program, Colombia.

Less than 1 % of the users served by the MIA program belonged to one of the ethnic groups identified in Colombian territory, and 2.1 % of the users resided in rural areas. Fig. 2 shows the geographical location of users. Fig. 2A shows the places of residence distributed throughout the Colombian territory. Fig. 2b shows that the five departments with the highest number of users were Antioquia (24.0 %), Bogotá D.C. (18.9 %), Valle del Cauca (8.9 %), Atlántico (8.5 %) and Norte de Santander (6.0 %), which together account for 66 % of all users.

### 3.2 Abortion result

Out of 1443 users followed, 1247 (86.4 %) had a confirmed complete abortion outcome through a pregnancy test or ultrasound. The remaining 196 (13.6 %) users needed an ultrasound to confirm the success of the abortion but didn't have a second follow-up.

Of the 1247 individuals who had an abortion and confirmed results, 1184 (94.9 %) had a successful MAB. The remaining 63 users (5.1 %) required procedural abortion. Table 1 displays user characteristics for those who completed abortion. The group of users who needed surgical intervention had a higher average age than those who completed the process with medication (28 vs. 25 years old; p = 0.002). No differences were observed in other sociodemographic characteristics.

### 3.3 Safety results

Table 2 showcases MAB procedure's safety results. Regarding common side effects, pain was the most common (89.1 %). Of the 1247 users who were followed up, 13.2 % reported fever, which was the most frequent potential sign of risk. Twenty-one (1.7 %) sought emergency department consultation, 20 of whom were in the complete abortion group. Three (0.2 %) other users required hospitalization and two (0.2 %) required intravenous antibiotics, all in the complete abortion group. In the complications results, four cases of infection (0.3 %) were reported, all of which were in the complete abortion group. No blood transfusions or deaths were reported.

**Table 2:** Effectiveness and safety outcomes of medical abortion in MIA users, Profamilia, Colombia, 2021–2022.

Results	Medication abortion results			
	Total	Complete abortion	Incomplete abortion	p-value
	n = 1247	n = 1184	n = 63	
	n (%)	n (%)	n (%)	
Common side effects				
Bleeding				
Yes	663 (53.2)	628 (53.0)	35 (55.6)	0.697^a^
No	584 (46.8)	556 (47.0)	28 (44.4)	
Pain				
Yes	1111 (89.1)	1057 (89.3)	54 (85.7)	0.377^a^
No	136 (10.9)	127 (10.7)	9 (14.3)	
Nausea and vomiting				
Yes	421 (33.8)	404 (34.1)	17 (27.0)	0.243^a^
No	826 (66.2)	780 (65.9)	46 (73.0)	
Diarrhea				
Yes	325 (26.1)	308 (26.0)	17 (27.0)	0.864^a^
No	922 (73.9)	876 (74.0)	46 (73.0)	
Chills				
Yes	449 (36.0)	422 (35.6)	27 (42.9)	0.245^a^
No	798 (64.0)	762 (64.4)	36 (57.1)	
Headache				
Yes	88 (7.1)	85 (7.2)	3 (4.8)	0.617^b^
No	1159 (92.9)	1099 (92.8)	60 (95.2)	
Potential warning wigns				
Fever				
Yes	165 (13.2)	157 (13.3)	8 (12.7)	0.898^a^
No	1082 (86.8)	1027 (86.7)	55 (87.3)	
Potential adverse events				
Emergency care				
Yes	21 (1.7)	20 (1.7)	1 (1.6)	0.951^b^
No	1226 (98.3)	1164 (98.3)	62 (98.4)	
Hospitalization				
Yes	3 (0.2)	3 (0.3)	0 (0.0)	0.689^b^
No	1244 (99.8)	1181 (99.7)	63 (100.0)	
Transfusion				
Yes	0 (0.0)	0 (0.0)	0 (0.0)	–
No	1247 (100.0)	1184 (100.0)	63 (100.0)	
Requirement of intravenous antibiotics				
Yes	2 (0.2)	2 (0.2)	0 (0.0)	0.744^b^
No	1245 (99.8)	1182 (99.8)	63 (100.0)	
Complications				
Infections				
Yes	4 (0.3)	4 (0.3)	0 (0.0)	0.813^b^
No	1243 (99.7)	1180 (99.7)	63 (100.0)	
Death, uterine rupture and hemorrhage				
Yes	0 (0.0)	0 (0.0)	0 (0.0)	–
No	1247 (100.0)	1184 (100.0)	63 (100.0)	
Time to start treatment				
Min-Max	0.0–38.0	0.0–38.0	0.0–22.0	–
Median/[IQR]	5.0/[3.0–7.0]	5.0/[3.0–7.0]	5.0/[3.0–7.0]	0.539^c^
1–7 days	974 (78.1)	922 (77.9)	52 (82.5)	0.677^a^
8–15 days	245 (19.7)	235 (19.9)	10 (15.9)	
>15 days	28 (2.2)	27 (2.3)	1 (1.6)	

a Chi Square Test of Independence.

b Fisher's exact test.

c Mann Whitney *U* test.

The time between consultation and the start of medication intake ranged from zero to 38 days, with a median of five days. 76.8 % of users received the abortion kit within the first seven days following consultation.

### 3.4 Satisfaction results

Satisfaction survey was answered by 194 of 3073 (5.3 %). Of these, 168 (86.6 %) were classified as promoters of the service and 26 (13.4 %) users who do not recommend the service. Seventy (36.1 %) users who responded to the survey were not effectively followed-up. Sensitivity analysis showed that there is no difference in the proportions of MAB success and having responded to the satisfaction survey or not (62.4 % vs 39.9 %; p = 0.158). Users who do not identify with any ethnicity and who have one or more previous pregnancies are more likely to participate in the satisfaction survey than those who do not respond, 99 % vs. 33.3 % (p = 0.001) and 50.4 % vs. 41.8 % (p = 0.015), (see Supplementary Table S1).

## 4 Discussion

This study aimed to describe the results of the first-trimester MAB service delivered via telemedicine at the non-governmental organization Profamilia.

One hundred eight (3.5 %) users were under 18 years old. The same age group, in European population studies, is approximately 0.4 % [33], while in American studies, it may range from 1 % to 10 % [34,35]. Profamilia's abortion telemedicine service has no age limit, although it is possible that certain pre-existing conditions could exclude certain people from accessing these services. A possibility may be lacking information and economic resources, or that pregnant women under 18 are subject to terms in health plans that violate their wishes for confidentiality and autonomy.

One concern is the low proportion of users who are residents of rural areas (2 %). One objective of remote healthcare services is to facilitate access to healthcare for people in remote regions. Geographical analysis shows that most service requests come from the central, northwest, and northeast regions. From the eastern and south-eastern regions of the country, as well as from the Pacific coast, users were scarce. This distribution may be related to the fact that these regions have higher poverty rates, fewer and lower quality educational institutions and, difficult access to computer and telecommunication technologies [36,37]. It is also observed that the five departments with the highest number of users are the five departments with the highest economic production in the country [38].

97.8 % of users began treatment within 15 days or less. This is favorable because the MIA program only accepts users within 12 weeks of pregnancy, ensuring that the abortion procedure will take place during the eligible weeks. Nonetheless, 2.2 % started treatment after 15 days. Delays in initiating treatment may necessitate the administration of additional doses of misoprostol or the performance of medical procedures [39].

We found that 94 % of the MAB was successful, a rate similar to those reported in studies that have examined telemedicine either separately or compared it with in-person care in countries such as the United States, Australia, or Mexico [16,19,23,25,34,[40], [41], [42], [43]].

Concerning MAB-related complications, some studies report incidence rates below 0.003 % [19,[23], [24], [25]], while others report values like those found in this study, close to 0.3 % [25,33,35,43]. It is essential to acknowledge that a few of the articles under consideration may diverge in their conceptualization of complications. Indeed, they include within the same category of events, whereas in this study, have been classified as potentially adverse.

Only 5 % of users voluntarily responded to the satisfaction survey and, the sensitivity analysis found statistically significant differences when comparing ethnicity and previous pregnancies. Thus, these results may not be representative of the population and may be affected by issues of internet access, privacy, or cultural or religious reasons, among others.

The follow-up rate was lower than that reported in other studies [22,23,40,43,44]. However, this is not an issue of concern since, according to WHO guidelines [1], the low rate of complications related to the use of mifepristone and/or misoprostol means that loss to follow-up cannot be an obstacle to accessing abortion services.

Limitations include inadequate characterization of self-reported secondary events and lack of integration of medical care information for emergency services or hospitalization. Additionally, users did not provide medical records or epicrises of their care for reported events. Finally, the satisfaction results may not accurately represent the entire user population because the sensitivity analysis indicates that the response rate to the survey may rise in the event of a successful medication procedure and side effects abscence.

MAB care delivered via telemedicine in developing countries can achieve standards of quality and safety comparable to those observed in developed countries. Consequently, the information presented is intended to promote confidence and enable rural or remote users to make informed decisions without having to rely on specialized health infrastructure. Further, it aims to inform political institutions, health service providers, and healthcare staff about the importance of implementing and strengthening information and access strategies.

## Ethical approval

The study received approval from the Profamilia Research Ethics Committee under Approval Record No. 5 of 2023. Additionally, participants provided written informed consent to participate in teleconsultation and receive MAB services, and they also consented to the use of their data for research purposes.

## Funding source

This research was supported by Profamilia (Colombia) and Ipas Latin America and the Caribbean. Ipas researchers have worked in the conceptualization and manuscript development.

## Declaration of competing interest

The authors declare that they have no known competing financial interests or personal relationships that could have appeared to influence the work reported in this paper.
