# Supplementary material for: First-trimester medication abortion via telemedicine: A retrospective cohort study
Source: Public Health Pract (Oxf). 2024 Aug 23;8:100539. doi: 10.1016/j.puhip.2024.100539 (PMC11415970; doi:10.1016/j.puhip.2024.100539)
Supplement: Multimedia component 1 [file mmc1.docx]

| **Supplementary table 1. Results of satisfaction surveys of MIA programme users and comparison between users who responded to the survey and those who did not, Profamilia, Colombia, 2021-2022** | | | | | | |
| --- | --- | --- | --- | --- | --- | --- |
| **Characteristics** | | | **Answered the satisfaction survey** | | |  |
|  |  |  | **No** | **Yes** | **p-value** |  |
|  |  |  | **n = 2,879** | **n = 194** |  |  |
|  |  |  | **n (%)** | **n (%)** |  |  |
| **Satisfaction (Net Promoting Score)** | | |  |  |  |  |
|  | Promoters (Score 9 or 10) | | **-** | 168 (86.6) | **-** |  |
|  | Non-Promoters (Score <9) | | **-** | 26 (13.4) |  |  |
| **Abortion Effectiveness result** | | |  |  |  |  |
|  | Successful medical abortion | | 1,063 (39.9) | 121 (62.4) | 0.158^c^ |  |
|  | Successful surgical abortion | | 60 (2.1) | 3 (1.6) |  |  |
|  | Missing | | 1,756 (70.0) | 70 (36.1) | - |  |
| **Potential warning signs** | | |  |  |  |  |
|  | Fever | |  |  |  |  |
|  |  | Yes | 172 (6.0) | 22 (11.3) | 0.289^c^ |  |
|  |  | No | 1,137 (39.5) | 112 (57.7) |  |  |
|  |  | Missing | 1,570 (54.5) | 60 (30.9) | - |  |
| **Potential adverse events** | | |  |  |  |  |
|  | Emergency care | |  |  |  |  |
|  |  | Yes | 46 (1.6) | 2 (1.0) | 0.232^c^ |  |
|  |  | No | 817 (28.4) | 83 (42.8) |  |  |
|  |  | Missing | 2,016 (70.0) | 109 (56.2) | - |  |
|  | Hospitalization | |  |  |  |  |
|  |  | Yes | 6 (0.2) | 1 (0.5) | 0.621^c^ |  |
|  |  | No | 857 (29.8) | 84 (43.3) |  |  |
|  |  | Missing | 2,016 (70.0) | 109 (56.2) | - |  |
|  | Transfusion | |  |  |  |  |
|  |  | Yes | 2 (0.1) | 0 (0.0) | 0.656^c^ |  |
|  |  | No | 858 (29.8) | 85 (43.8) |  |  |
|  |  | Missing | 2,019 (70.1) | 109 (56.2) | - |  |
|  | Intravenous antibiotics | |  |  |  |  |
|  |  | Yes | 2 (0.1) | 0 (0.0) | 0.655^c^ |  |
|  |  | No | 844 (29.3) | 84 (43.3) |  |  |
|  |  | Missing | 2,033 (70.6) | 110 (56.7) | - |  |
| **Complications** | | |  |  |  |  |
|  | Infections | |  |  |  |  |
|  |  | Yes | 6 (0.2) | 0 (0.0) | 0.432^c^ |  |
|  |  | No | 1,303 (45.3) | 134 (69.1) |  |  |
|  |  | Missing | 1,570 (54.5) | 60 (30.9) | - |  |
| **Sociodemographic** | | |  |  |  |  |
|  | Age | |  |  |  |  |
|  |  | Min-Max | 13-47 | 15-46 |  |  |
|  |  | Median/[IQR] | 25 (22-29) | 25 (21-30) | 0.705^a^ |  |
|  |  | < 18 years | 0 (0.0) | 6 (3.6) | 0.640^c^ |  |
|  |  | 18–24 years | 12 (46.2) | 78 (46.4) |  |  |
|  |  | 25–34 years | 13 (50.0) | 67 (39.9) |  |  |
|  |  | ≥ 35 years | 1 (3.9) | 17 (10.1) |  |  |
|  | Area of residence | |  |  |  |  |
|  |  | Urban | 2,813 (97.7) | 191 (98.4) | 0.587^c^ |  |
|  |  | Rural | 61 (2.1) | 3 (1.6) |  |  |
|  |  | Missing | 5 (0.2) | 0 (0.0) | - |  |
|  | Socioeconomic status | |  |  |  |  |
|  |  | Low (strata 1 and 2) | 2,433 (84.5) | 172 (88.7) | 0.254^c^ |  |
|  |  | Middle (strata 3 and 4) | 428 (14.9) | 21 (10.8) |  |  |
|  |  | High (strata 5 and 6) | 13 (0.4) | 1 (0.5) |  |  |
|  |  | Missing | 5 (0.2) | 0 (0.0) | - |  |
|  | Level of education | |  |  |  |  |
|  |  | Up to primary school | 30 (1.0) | 1 (0.5) | 0.424^c^ |  |
|  |  | Up to secondary school | 2,334 (81.1) | 173 (89.2) |  |  |
|  |  | Two- or three-year associate degree | 150 (5.2) | 7 (3.6) |  |  |
|  |  | Undergraduate/Postgraduate degree | 138 (4.8) | 13 (6.7) |  |  |
|  |  | Missing | 227 (7.9) | 0 (0.0) | **-** |  |
|  | Healthcare affiliation regimen | |  |  |  |  |
|  |  | Contributory | 2,082 (72.3) | 138 (71.1) | 0.187^c^ |  |
|  |  | Subsidized | 435 (15.1) | 37 (19.1) |  |  |
|  |  | Special regime | 357 (12.4) | 18 (9.3) |  |  |
|  |  | Missing | 5 (0.2) | 1 (0.5) | - |  |
|  | Marital status | |  |  |  |  |
|  |  | Single/Separated/Divorced/Widowed | 2,611 (90.7) | 179 (92.3) | 0.505^c^ |  |
|  |  | Married/De facto union | 263 (9.1) | 15 (7.7) |  |  |
|  |  | Missing | 5 (0.2) | 0 (0.0) | - |  |
|  | Ethnicity | |  |  |  |  |
|  |  | None | 2,851 (99.0) | 1 (33.3) | **<0.001^c^** |  |
|  |  | Other (Afro-descendant, Raizal) | 23 (0.8) | 1 (33.3) |  |  |
|  |  | Missing | 5 (0.2) | 1 (33.4) | - |  |
| Clinical | |  |  |  |  |  |
|  | Gestational age | |  |  |  |  |
|  |  | Min-Max | 2.5-12.4 | 4-8.5 |  |  |
|  |  | Median/[IQR] | 6.4 (5.5-7.3) | 6.3 (5.6-7.3) | 0.716^a^ |  |
|  |  | ≤ 8 weeks | 2,545 (88.5) | 169 (87.1) | 0.562^b^ |  |
|  |  | > 8 weeks | 331 (11.5) | 25 (12.9) |  |  |
|  | Previous pregnancies | |  |  |  |  |
|  |  | Yes | 1,452 (50.4) | 81 (41.8) | **0.015^c^** |  |
|  |  | No | 1,399 (48.6) | 112 (57.7) |  |  |
|  |  | Missing | 28 (1.0) | 1 (0.5) | **-** |  |
| ^a^ Mann–Whitney U test | | |  |  |  |  |
| ^b^ Chi-Square Test of Independence | | |  |  |  |  |
| ^c^ Fisher’s Exact test | | |  |  |  |  |
